# Automatic Suppression of Intense Monochromatic Light in Electro-Optical Sensors

**DOI:** 10.3390/s121014113

**Published:** 2012-10-19

**Authors:** Gunnar Ritt, Bernd Eberle

**Affiliations:** Fraunhofer Institute of Optronics, System Technologies and Image Exploitation (IOSB), Gutleuthausstr. 1, 76275 Ettlingen, Germany

**Keywords:** laser dazzling, sensor protection, spatial light modulator, wavelength multiplexing

## Abstract

Electro-optical imaging sensors are widely distributed and used for many different tasks. Due to technical improvements, their pixel size has been steadily decreasing, resulting in a reduced saturation capacity. As a consequence, this progress makes them susceptible to intense point light sources. Developments in laser technology have led to very compact and powerful laser sources of any wavelength in the visible and near infrared spectral region, offered as laser pointers. The manifold of wavelengths makes it difficult to encounter sensor saturation over the complete operating waveband by conventional measures like absorption or interference filters. We present a concept for electro-optical sensors to suppress overexposure in the visible spectral region. The key element of the concept is a spatial light modulator in combination with wavelength multiplexing. This approach allows spectral filtering within a localized area in the field of view of the sensor. The system offers the possibility of automatic reduction of overexposure by monochromatic laser radiation.

## 1. Introduction

Imaging sensors operating in the visible, near-infrared or thermal infrared are widely distributed devices. They are used for a vast number of applications, such as environmental monitoring or workpiece inspection. Advances in production technology have led to constantly shrinking sensor sizes along with an increasing number of pixels. This trend is mainly driven by the consumer market, where portable digital devices have moved to smaller form factors and thus require smaller imaging sensors. For instance, imaging sensors with pixels measuring only 1.4 μm are available [1]. Unfortunately, this miniaturization can cause a loss of performance, for example a lower dynamic range of the sensors [2]. Furthermore, the maximum signal that can be recorded by the sensor depends on the full-well capacity of a pixel. In general, small pixels offer a lower full-well capacity [3] compared to sensors with larger pixel size and thus can be saturated more easily by intense light sources. While this may not represent a problem for consumer video cameras, it can have a serious impact for sensors used in security or defence applications. The intentional dazzling of sensors by intense light sources, in particular by high-power lasers, is a conceivable threat.

More than 50 years after the invention of the laser, the technological progress has made very compact but nevertheless powerful laser sources possible [4]. These days, hand-held laser pointers emitting in the visible spectral region with output powers up to one watt are available freely on the market. In the visible spectral region, lasers of any wavelength can be produced. The consequence of that increasing availability of such laser sources is, however, the increase in misuse of such devices and represents a serious hazard. In civil environments, particularly aircraft crews as well as motorists are considered to be candidates for dazzling attacks [5,6]. In such cases, the disturbance of the vision can be catastrophic. Besides that, not only for the human eye but also for electro-optical sensors used in autonomous or surveillance systems, dazzle is a severe problem [7]. An adequate protection against dazzling for these sensors is desirable.

Current laser protection measures typically involve the use of conventional optical filters based on absorption or interference effects. Unfortunately, these devices only work in a limited wavelength range, and moreover, they may introduce a strong colour distortion in the visible spectral range. The colour distortion can be minimized or reduced by making use of narrow-band notch filters, which is at the expense of broadband safety. Therefore, sophisticated anti-dazzling protection concepts which do not influence the system performance are required. A perfect protection measure for electro-optical sensors should fulfil various requirements, among them:

- Broadband protection in the complete operating wavelength range of the sensor. Ideally, the protection measure is able to protect against several different wavelengths at the same time.
- Sufficient attenuation of dazzling laser light, while offering a high transmittance for non-dazzling radiation.
- No colour distortion is introduced in the sensor image due to the protection measure.
- The protection measure acts only on those parts of the field of view where dazzling radiation occurs. Areas where no dazzling is present are not affected.
- Protection against monochromatic dazzling sources (both continuous wave and pulsed lasers) as well as broadband dazzling sources (e.g., the sun, high power LEDs).

Numerous kinds of approaches were discussed in literature regarding realization concepts for laser protection. A short review of these technologies is given by Svensson and co-workers [6]. They distinguish three types of protection measures: (a) *static protection*, (b) *active protection* and (c) *self-activating protection*. Static protection measures, such as fixed line filters, offer a very high attenuation, but only for a limited wavelength range. Active systems suffer from the disadvantage of a finite response time and are therefore not able to protect against single laser pulses. A self-activating protection seems to be the most promising technology. In the last decades, optical power limiters were investigated as broadband laser protection. An optical power limiter is a device offering a high transmittance for low light intensities, while showing a decreased transmittance when exposed to high optical intensities. Several reviews on optical power limiters can be found in literature [8,9]. Usually, optical power limiters are not usable as protection against laser dazzling because the activation threshold is far too high. They are intended to protect the eye or electro-optical systems against damage by laser radiation. However, in recent publications a dynamic sunlight filter based on this kind of technology was presented [10,11]. The maximum attenuation is stated to be 20dB and is intended to be increased to more than 30dB. As described later in this publication, the attenuation of such a filter has to be improved for the use in realistic situations.

In summary, it can be said that none of the protection technologies available today is able to fulfil all the requirements stated before. Here, we present a novel concept for automatic suppression of intense monochromatic light in electro-optical sensors. It is based on the recognition of dazzling light and its suppression, while low power radiation is nearly unaffected. In order to realize this concept, we made use of a spatial light modulator (SLM) in combination with wavelength multiplexing as the key elements. Such an active concept is definitely attractive, since the proliferation of compact continuous wave (cw) laser pointers is very high and largely uncontrolled.

## 2. Theoretical Considerations

One of the requirements for a potential protection measure is *sufficient attenuation* of dazzling light. The attenuation required to suppress overexposure of the sensors depends on the parameters of the sensor (e.g., exposure time) and the laser (e.g., output power) as well as on the environmental conditions (e.g., distance from the laser to the sensor, atmospheric extinction). Here, a simple estimate of the attenuation needed shall be given, assuming a standard situation.

### 2.1. Estimate of the Saturation Irradiance

We assume that a pixel of the sensor is illuminated with an irradiance *E*. The number of photons *μ*_P_ arriving at a pixel with the area *A* during the exposure time *t*_exp_ is given by [12]:

(1) $$\mu_{\text{P}}=\frac{EAt_{\exp}}{hc/\lambda }$$

where *h* = 6.626 × 10^−34^J/s is the Planck constant, *c* = 2.99792458 ×·10^8^m/s the speed of light and *λ* the wavelength of the light. The number of photoelectrons *μ*_e_ generated in the pixel is determined by the quantum efficiency *η*:

(2) $$\mu_{\text{e}}=\eta \cdot \mu_{\text{P}}$$

For the saturation irradiance *E*_sat_, the number of photoelectrons *μ*_e_ equals the saturation capacity *μ*_e.sat_. The saturation capacity is usually lower than the full well capacity *C* of the pixel [12], but for simplicity, we equal both values. The saturation irradiance can then be estimated by:

(3) $$E_{\text{sat}}=\frac{C\cdot hc/\lambda }{\eta At_{\exp}}$$

If the full well capacity for the camera sensor in use is not known, it can be calculated by a rule of thumb stated by Holst and Lomheim [3]:

(4) $$C=1500\frac{{\text{e}}^{-}}{\mu {\text{m}}^{2}}\cdot A$$

For the camera used in our setup (VRmagic VRmC-12 Pro) the area of a pixel is *A* = 6 μm^2^ and the quantum efficiency *η* ≈ 0.35. For a typical exposure time *t*_exp_ = 20 ms we calculate a saturation irradiance of *E*_sat_ = 8μW/cm^2^ for light of wavelength *λ* = 532 nm using Equations (3) and (4).

### 2.2. Estimate of the Laser Irradiance

The main parameters to characterize the propagation of a laser beam are: beam diameter *d*_0_ at the exit port, output power *P*_0_ and full angle divergence *Φ*. The beam diameter in a distance *z* from the laser exit port is given by:

(5) $$d(z)=\sqrt{{d_{0}}^{2}+\Phi^{2}z^{2}.}$$

If we assume a Gaussian beam profile, the power *P*_ap_ entering the entrance aperture of an optical system with diameter *D* located at a distance *z* is [13]:

(6) $$P_{\text{ap}}(z)=P_{0}\cdot \exp(-\sigma z)\cdot \left(1-\exp\left(-2\frac{D^{2}}{d^{2}(z)}\right)\right),$$

where *σ* is the extinction coefficient of the atmosphere.

If the optical system is located far away from the laser, then *d*(*z*) ≫ *D*. Thus, plane waves of uniform intensity distribution are falling onto the aperture of the optical system. In this case, an Airy diffraction pattern is formed at the focal plane (focal length *f*) and the peak irradiance at the focal point *E*_peak_ can be estimated by [14]:

(7) $$E_{\text{peak}}=\frac{P_{\text{ap}}\cdot \frac{\pi }{4}D^{2}}{\lambda^{2}f^{2}}$$

A more accurate formula for the peak irradiance is given by Haskal, considering the truncation of a Gaussian beam profile by a circular aperture [15]:

(8) $$E_{\text{peak}}=\frac{\pi P}{2\lambda^{2}F^{2}}\frac{{\left(1-\exp(-1/\nu^{2})\right)}^{2}}{1/\nu^{2}}$$

Here, *P* = *P*_0_ × exp(-*σz*) is the total laser power at the distance *z*, *F* = *f*/*D* the f-number of the optical system and *ν* = *d*(*z*)/*D* the so-called truncation factor. Additionally, we can account for the optics transmission by including a factor *T* in Equations (7) and (8).

**Figure 1.**
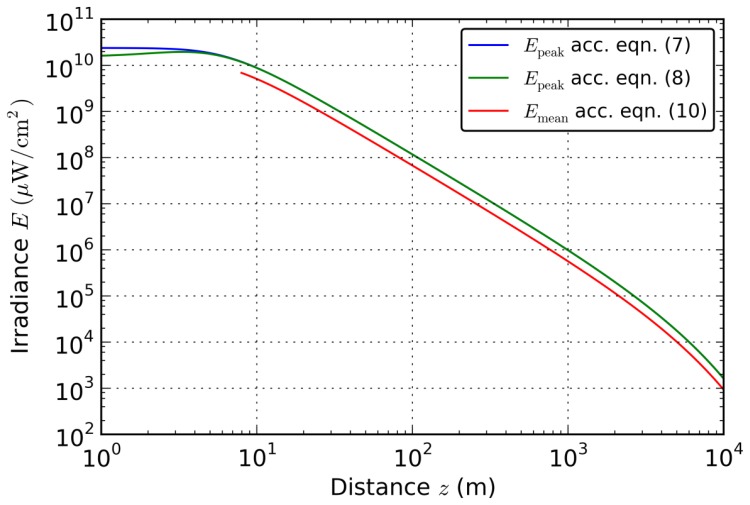
Comparison of Equations (7), (8) and (10) for the peak irradiance and mean irradiance in the focal plane of an optical system, respectively.

A comparison of both equations for the peak irradiance is shown in Figure 1 (blue and green curves). For this plot, we used the following values corresponding to the parameters of the laser used for testing our protection concept (see Section 4):

- Laser: *P*_0_ = 36 mW, *d*_0_ = 3 mm, *Φ* = 1 mrad, λ = 532 nm
- Optical system: *D* = 5 mm, *f* = 50 mm, *T* = 0.24
- Atmosphere: *σ* = 0.2 km^−1^ (corresponding to a visibility of 19.6 km according to Koschmieder's law [16])

As can be seen from Figure 1, major differences only occur for distances below 5m. This is mainly because Equation (7) assumes uniform illumination of the complete optics aperture. For distances below 5m, uniform illumination is not realistic because the beam diameter of the laser is smaller than the optical system's aperture. This would result in a wider diffraction pattern and therefore in a lower peak irradiance. For larger distances there is no difference between the results of the Equations (7) and (8).

Since a pixel of the sensor possesses a finite area, instead of the peak irradiance the mean irradiance over the pixel size has to be calculated. The power impinging onto a circular disc with radius *r* around the centre of the Airy distribution (*encircled power*) is given by [14]:

(9) $$P_{\text{enc}}=P_{\text{ap}}\left(1-{\text{J}}_{0}^{2}(ka\sin\Theta )-{\text{J}}_{1}^{2}(ka\sin\Theta )\right)$$

Here, *P*_ap_ is the radiation power entering the optics (see Equation (6)), *k* = 2π/λ the wavenumber, *a* = *D*/2 the radius of the entrance aperture and θ = *r*/*f* the half angle under which the circular disc is seen from the location of the diffracting aperture (entrance aperture). J_0_ and J_1_ are Bessel functions of the first kind.

Usually a sensor pixel exhibits a rectangular area. Thus, the encircled power has to be corrected in order to obtain the *ensquared power*. The maximum of the correction factor is about 1.073 [17] and the mean irradiance at the sensor pixel is:

(10) $$E_{\text{mean}}=\frac{P_{\text{enc}}}{A}\cdot 1.073,$$

which is shown by the red curve in Figure 1. Since the calculation relies on the assumption of an Airy diffraction pattern, the plot starts at a distance value where the difference between Equations (7) and (8) is below one percent. The values of the mean irradiance are a factor of 1.4 lower than the values of the peak irradiance.

From Figure 1 we can deduce that for distances below 1,000m the calculated peak irradiance exceeds the calculated saturation irradiance (8 μW/cm^2^) by a factor >10^5^. From this result, we conclude that effective dazzling suppression needs an attenuation of at least five orders of magnitude.

## 3. Experimental Setup

The application of spatial light modulators as a protection measure against dazzling light sources was already described earlier by Tomilin and Danilov, but no experimental results were given [18]. In previous investigations, we demonstrated successfully such an approach [19]. The applicability of this method was significantly improved by the implementation of wavelength multiplexing, which allows now spatial and spectral filtering in contrast to [18].

Wavelength multiplexing is a technique which was introduced by Koester to improve the quality of images transferred through optical fibre bundles. The idea was to transmit the various wavelengths from a given object point through a number of different fibres [20]. It was realized by placing a *double Amici prism* (or *direct vision prism*) in front of the input optical system of a fiberscope, which imaged the object onto the entrance facet of the fibre bundle. Thus, the light from a given object point was spectrally broken down and then transmitted through the fibre bundle. A corresponding dispersing element was placed at the exit end of the fibre bundle to reverse the dispersion. Therefore, undesired transmission errors of the fibre bundle, for example caused by broken fibres and the facet structure, were reduced.

**Figure 2.**
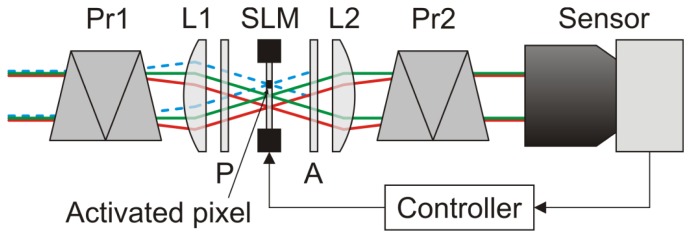
Sensor protection concept using wavelength multiplexing by means of two direct vision prisms (Pr1/Pr2: direct vision prisms; L1/L2: lenses; SLM: spatial light modulator; P/A: polarizers). Due to the spectral dispersion, the activation of the single SLM elements attenuates only a narrow spectral band (blue dashed rays) of the incident light beam, whereas all remaining wavelengths from the incident beam can pass through the optical arrangement unaffected (red and green rays). This setup allows for combined spatial and spectral filtering of monochromatic light sources whatever the wavelength.

Our first experimental setup making use of a spatial light modulator in combination with the wavelength multiplexing technique is shown in Figure 2. Here, a transmissive liquid crystal SLM (Holoeye LC2002) in combination with two polarizers (denoted as P and A in Figure 2) is used as an intensity modulator. In order to be able to filter light only in localized areas of the sensor's field of view, the SLM is located at the intermediate focal plane of a 1:1 Keplerian telescope (formed by lenses L1 and L2). Before and behind the telescope, two identical direct vision prisms (Pr1 and Pr2) are inserted into the optical beam path to implement the wavelength multiplexing.

Light beams entering the setup are spectrally broken down by the first prism. Each object point of a distant scene forms a wavelength spectrum at the intermediate focal plane of the telescope. Due to the different angles each object point is seen, the corresponding wavelength spectrum is formed at a slightly different position on the SLM. In order to restore the object scene, the dispersion is reversed by means of the second prism behind the telescope. As depicted in Figure 2, the transmission of a specific spectral range of the light beam (here represented by the blue dashed lines) can be blocked by activating the appropriate pixels of the SLM. All the remaining wavelengths can pass the optical arrangement unaffected. Such a setup allows for combined spatial and spectral filtering of monochromatic light sources and can be used particularly as a protection measure against laser dazzling which ideally suppresses only the threatening laser light, while not affecting the non-threatening radiation from the scene.

While this setup was well suited to demonstrate the proof of principle, it suffered from its low transmittance. This was mainly caused by the liquid crystal SLM and the polarizers necessary to use the SLM as an intensity modulator. The direct vision prisms offer high transmittance but need a large amount of space (106 mm × 30 mm × 30 mm) which would impede the construction of compact and lightweight systems. To overcome these limitations, an improved setup based on a *digital micromirror device* (DMD) was developed. A DMD consists of an array of micromirrors that are tiltable between two stable states, forming different angles with respect to the normal of the array. Due to the reflective design, there is no need for polarized light, resulting in a higher transmittance.

**Figure 3.**
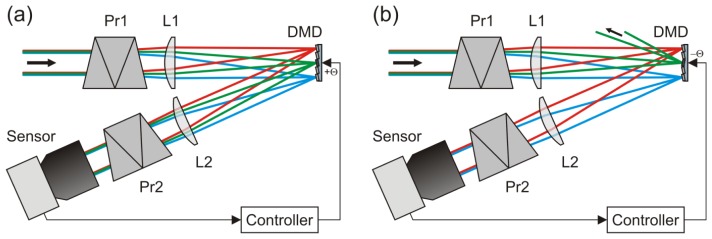
Sensor protection concept using a DMD. (**a**) Operation mode for regular imaging: all the micromirrors are tilted towards the sensor. (**b**) Operation mode with high attenuation for dazzling light: the micromirrors which are exposed with dazzling laser light (here: the green rays) are tilted away from the sensor. Thus, the dazzling light will be strongly attenuated in the regular imaging path.

A schematic view of the optical setup using a DMD is shown in Figure 3. Usually, this setup would be operated in such a way that the light is directed towards the sensor by tilting all micromirrors to the +θ-state [Figure 3(a)]. In the case of dazzling laser light arriving at the sensor (here: the green rays in the figure), the controller toggles just these micromirrors to the -θ-state which are exposed with dazzling light [Figure 3(b)]. Thus, the dazzling light is reflected out of the beam path, whereas the non-dazzling light can still reach the camera.

For our application, we chose the DLPDiscovery4100 kit from Texas Instruments comprising the 0.7″ XGADMD. This DMD offers 1,024 × 768 micromirrors with a pitch of 13.68 μm. Each mirror can be switched from a +12° to a −12° state; the axis of rotation is oriented at an angle of 45° with respect to the edges of the array. The DMD efficiency for wavelengths between 420 nm and 700 nm is specified to be 68%, which includes the transmission of the protection window (∼97%), the fill factor (∼92.5%), the mirror reflectivity (∼88%) and the diffraction efficiency (∼86%). This value is defined as the amount of light that is reflected specularly by the array.

**Figure 4.**
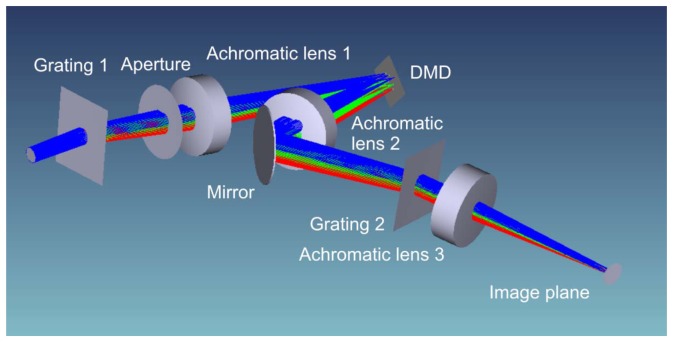
Optical layout of the sensor protection concept. A digital micromirror device (DMD) is located in the intermediate focal plane of a Keplerian telescope (Achromatic lens 1 and 2). Wavelength multiplexing is implemented by the use of two diffraction gratings (Grating 1 and 2). Grating 1 is aligned so that the spectral separation takes place in a plane perpendicular to the plane of reflection at the DMD.

The optical layout of the improved setup is shown in Figure 4. Since the DMD works in reflection, the beam path is folded (deviation angle of 24° to the normal of the DMD surface). The DMD is placed at the intermediate focal plane of a Keplerian telescope formed by two achromatic lenses with a focal length of 75 mm (Achromatic lens 1 and 2). A second folding mirror behind the telescope is used to realize a more compact footprint of the complete setup.

In order to further compact the setup, we decided to use gratings instead of prisms to implement the wavelength multiplexing, but on the cost of transmittance. We use two blazed diffraction gratings with 300 grooves/mm (Grating 1 and 2) which offer an absolute efficiency of more than 60% for wavelengths in the range from 450 nm to 700 nm. The absolute efficiency is defined by the ratio of the energy diffracted into the preferred order to the total incident energy. As can be seen in Figure 4, the gratings are aligned so that the spectral separation takes place in a plane perpendicular to the plane of reflection at the DMD.

A third lens (Achromatic lens 3) with a focal length of 50mm forms the final image. The focal plane of the achromatic lens 2 and the plane of the DMD mirrors do not coincide. As a consequence, the final image plane is tilted with regard to the optical axis. In our setup, we use a VRmC-12 Pro CMOS camera from VRmagicGmbH (Mannheim, Germany) that is aligned accordingly, which is known as the principle of Scheimpflug.

## 4. Results of the Sensor's Dazzling Suppression Capabilities

The main characteristics (*i.e.*, the system transmission and attenuation) of the setup described above were quantified by two different measurements: (1) The attenuation for monochromatic light turned out to be 48dB at a wavelength of 532nm. (2) The broadband system transmission was measured to be 24% using a halogen cold-light source.

**Figure 5.**
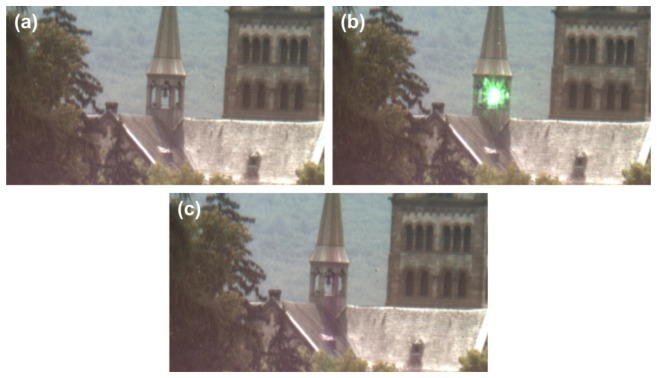
View of an urban scene imaged with an optical system as presented in Figure 4. (**a**) View of the undisturbed scene. (**b**) Disturbed scene: Laser radiation originating from the steeple dazzles the camera. (**c**) View of the scene while the filtering is switched on. As a side effect a vertically arranged colour distortion slightly is recognisable, but all geometrical details are visible.

The results of a field trial are shown in Figure 5. The urban scene shows the roof part of a church, where a retroreflector was mounted at a column of the small steeple (distance: 412 m). A laser (continuous wave, wavelength: 532 nm, output power: 36 mW, full angle divergence: 1 mrad), which was placed next to the sensor in the laboratory, served as dazzling light source. The irradiance from the retroreflected laser beam at the entrance aperture of our optical system was approximately 10 μW/cm^2^ (assuming Gaussian beam propagation and an atmospheric extinction coefficient of 0.2 km^−1^).

As soon as the laser was switched on, dazzling occurred. As can be seen in Figure 5(b), the laser glare blurred the scene in the vicinity of the steeple bell completely. When the control loop of the system was activated, the micromirrors of the DMD corresponding to the overexposed camera pixels were toggled and the dazzling laser light was removed from the regular beam path. Figure 5(c) shows the undisturbed scene, which is now completely observable again; only a slight colour distortion within a narrow vertical stripe is recognizable in the camera image.

## 5. Implementation of the Control Loop

The task of the controller indicated in Figure 3 is to analyse steadily the camera images for dazzling and to drive the DMD automatically according to the actual situation. In case of dazzling laser light, the controller has to toggle the appropriate micromirrors of the DMD in order to suppress the overexposure. The activation of the correct micromirrors depends (1) on the direction from where dazzling originates and (2) on the wavelength of the dazzling laser.

The direction from where the dazzling originates is delivered directly from the camera image. However, the wavelength of the dazzling laser is a priori not known. This means that the exact location where the dazzling light is focused onto the DMD is not known. In the first instance, only information on the appropriate columns of micromirrors is given since the dispersion of light due to the grating takes place in a vertical direction.

### 5.1. Wavelength Estimation from RGB Colour Values

For the case of a colour camera to be protected, the information on the dazzling wavelength can be deduced directly from the colour information contained in the camera images. The overexposed regions on the detector are surrounded by unimpaired pixels which contain the necessary information about the searched wavelength. For instance, in the situation shown in Figure 5(b) an observer could immediately say that some green laser light affects the sensor. A good estimation of the dazzling wavelength can be derived by an analysis of the colour values of the non-saturated contour pixels surrounding the overexposed area.

**Figure 6.**
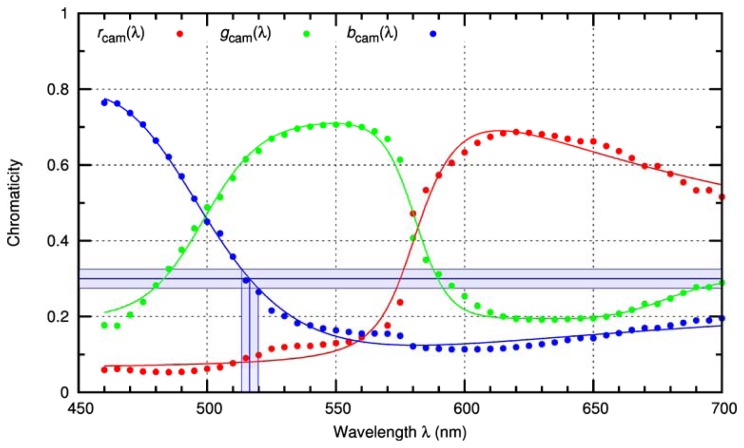
Colour response curves of the camera used in the optical setup (VRmagicVRmC-12 Pro). The dots in the plot show measured chromaticity values for monochromatic light. The solid curves are fit curves to the measurement data.

The image exploitation algorithm implemented to derive the contour line is described in Section 5.2 in more detail. Assuming that the contour is already defined, the mean colour values *R*, *G* and *B* of the pixels along the contour are calculated. In a first step, these values are normalized to get the intensity independent chromaticity values *r* = *R*/(*R* + *G* + *B*), *g* = *G*/(*R* + *G* + *B*), *b* = *B*/(*R* + *G* + *B*). These values are compared with the camera's colour response curves *r*_cam_(λ), *g*_cam_(λ) and *b*_cam_(λ) for red, green and blue light, respectively. The response curves of the camera are plotted in Figure 6.

To account for uncertainties in the measured RGB values, for example by noise, the wavelength estimation is performed using chromaticity intervals [*r* − Δ, *r* + Δ], [*g* − Δ, *g* + Δ] and [*b* − Δ, *b* + Δ]. Initially, the uncertainty value Δ is arbitrarily set to 0.025. Then, those wavelengths are determined at which the camera's response curve is contained within the corresponding chromaticity interval.

As an example for *b* = 0.3, this procedure is illustrated in Figure 6 for the blue chromaticity interval [*b* − Δ, *b* + Δ] and the camera response curve *b*_cam_(λ) for blue light.

By doing this, three sets of wavelengths are provided—one for each colour:

(11a) $$W_{\text{red}}=\{\lambda |r-\Delta \leq r_{\text{cam}}(\lambda )\leq r+\Delta \}$$

(11b) $$W_{\text{green}}=\{\lambda |g-\Delta \leq g_{\text{cam}}(\lambda )\leq g+\Delta \}$$

(11c) $$W_{\text{blue}}=\{\lambda |b-\Delta \leq b_{\text{cam}}(\lambda )\leq b+\Delta \}$$

Subsequently, the intersection of all three sets of wavelengths is computed:

(12) $$W=W_{\text{red}}\cap W_{\text{green}}\cap W_{\text{blue}}$$

The set *W* most likely contains the laser wavelength since the values of the set *W* are the possible wavelengths associated with the chromaticity values *r*, *g* and *b*. If the set *W* is empty after the first iteration of the algorithm, the computation is repeated with a value Δ increased by 0.025 until *W* contains values. In conclusion, this method provides not a single wavelength but wavelength bands.

**Figure 7.**
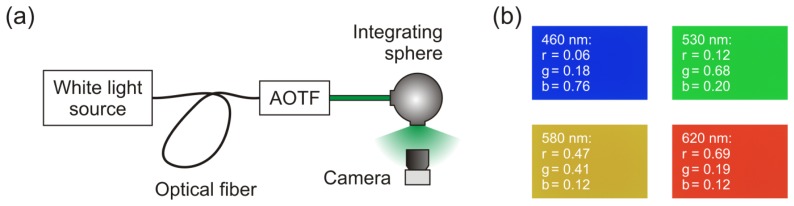
(**a**) Experimental setup used to investigate the accuracy of the algorithm to estimate laser wavelength with the help of a colour camera. AOTF: Acousto-optical tunable filter. (**b**) Some examples of images taken with the camera under illumination with a specific wavelength and the corresponding chromaticities *r*, *g* and *b*.

In order to estimate the capability of this approach for our problem of determining the wavelengths of dazzling lasers, we performed a series of measurements. The experimental setup for these measurements is shown in Figure 7(a). A coherent white light source (Koheras SuperK Extreme) equipped with an acousto-optical tunable filter (AOTF) was used to produce narrow-band radiation adjustable in the spectral range from 460nm to 700nm. With the help of an integrating sphere, the camera sensor was successively illuminated homogeneously with a large number of different wavelengths. For each wavelength, an image was taken with the camera. Figure 7(b) shows four examples for such images. Please note that the camera was operated without any colour calibration. The exposure time was changed during the measurements to adjust the signal to be around 85% of the dynamic range of the camera for each measurement. Measurements with wavelengths below 460nm were not possible due to the low power of the white light source for these wavelengths.

**Figure 8.**
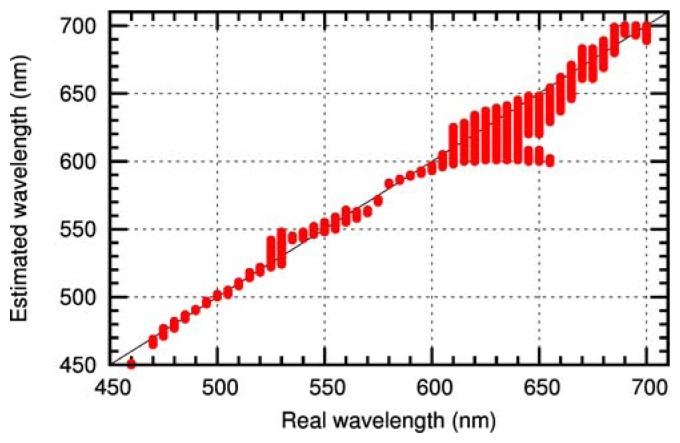
Accuracy of estimated laser wavelengths evaluated by the RGB values of a colour camera.

Figure 8 shows the accuracy of this method as obtained for the camera used in the experimental setup. In the graph, the set of possible wavelengths *W* (see Equation (12)) estimated by the algorithm is plotted as a function of the input wavelength. The accuracy of the estimated wavelength is quite good for wavelengths up to 600nm. For wavelengths above 600nm the uncertainty in wavelength estimation increases, resulting from the flatness of the response curves (see Figure 6). As a result, the control loop of the sensor has to be adapted to the uncertainty in wavelength estimation. This is achieved by toggling a corresponding larger number of micromirrors.

### 5.2. Image Analysis to Deduce RGB Colour Values

**Figure 9.**
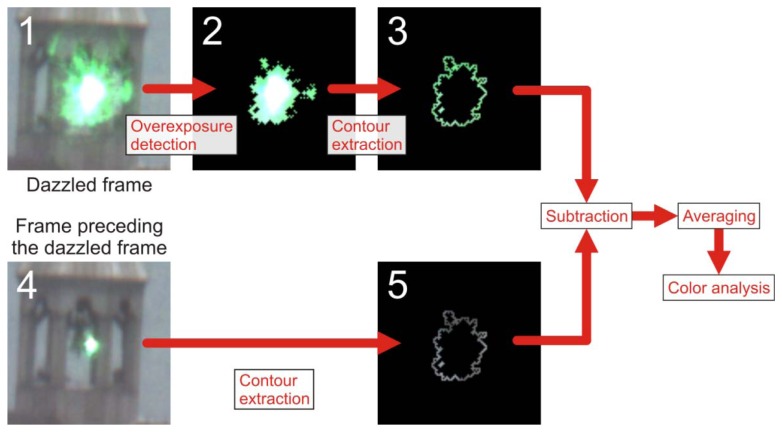
Flowchart of the image analysis procedure for the estimation of the dazzling wavelength. First, the overexposed pixels (see Image 2) of a dazzled frame (see Image 1) are determined. The colour of the contour line (see Image 3) is ready to be analysed after the subtraction of the background (see Image 5). The frame preceding a dazzled frame (see Image 4) is used to determine the background light.

The wavelength analysis method described in Section 5.1 is based on the knowledge of the RGB values of the dazzled area. A flowchart of the procedure of analysis of the camera images is depicted in Figure 9. In order to decide whether dazzling has occurred or not, a first step is to detect the overexposed pixels in each camera image. This is done by a simple thresholding operation (threshold value: 254) in all three RGB bands. A pixel with a value above threshold within at least one of the RGB bands is considered as overexposed. The result of the thresholding operation applied to Image1 of Figure 9 is shown in Image 2 of Figure 9. Since saturated pixels contain no useful RGB information, the neighbouring pixels forming a contour line around the overexposed area will be extracted and used for further analysis (see Image 3 of Figure 9).

At this point, one has to take into account that the background light influences the result of the analysis of the RGB values and has to be removed for exact wavelength estimation. For this purpose, we make use of the camera frame preceding the dazzled frame (see Image 4 of Figure 9). This is appropriate as long as there are only moderate changes in the illumination conditions between the acquisitions of two subsequent camera images. The according contour line in the preceding image is shown in Image 5 of Figure 9. As a last step, the background corrected pixel values of the contour line are subsequently averaged and the resulting triple (*R*, *G*, *B*) is analysed to estimate the laser wavelength as described in Section 5.1.

### 5.3. Control Loop Response Time—General Restrictions

A technical characterization of the system would involve a specification of the system's response time. For our sensor system, the speed of the control loop is mostly limited by the software algorithm since the computation is the most time consuming part. The computation involves the detection of overexposure due to laser irradiation, the estimation of the laser wavelength as described in Section 5.1 and the creation of an appropriate binary pattern to drive the DMD. As response time to react on laser dazzling, we define the time between the occurrence of dazzling and the activation of the DMD.

A fixed value for the response time cannot be stated since the computation time strongly depends on the complexity of the scene. In the case of a single dazzle laser, the overall computing time is well below 20ms using a computer with up-to-date central processing unit (CPU) and graphics processing unit (GPU). For such a simple situation, the algorithm operates in real-time without the loss of camera frames. The switching rate for the full DMD array is higher than 300Hz and does not affect the speed of the control loop. During the computation, the camera already captures one or more new images depending on the integration time. Therefore, at least two dazzled camera images will occur.

The computational process depends on the complexity of the scene. We can distinguish between different scenarios: (a) Multiple laser sources are directed to the sensor from different directions with separated glares in the camera image. (b) Multiple laser sources with different wavelengths are directed to the sensor from the same position or from positions close to each other. In this case, their glares overlap in the camera image. (c) Mix of situation (a) and situation (b).

In the case of situation (a), the computing time increases with the number of dazzle lasers since the estimation of the individual wavelengths is a sequential process. This process does not depend on the particular wavelength.

Situation (b) gets demanding when more than three different wavelengths will be used or the spectral separation of the wavelengths cannot be resolved by the RGB colour analysis. With increasing number of wavelengths, the brightness of the corresponding columns in the camera image will be reduced.

Case (c) may be quite challenging. Depending on the scenario, the RGB colour analysis might not be sufficient to deal with the situation and additional measures have to be used. One possible measure, for instance, is using in addition a compact spectrometer to optimize the wavelength estimation. The procedure in case of laser dazzling would be as follows:

1. The image analysis algorithm roughly estimates the occurring wavelengths on the basis of the colour values (e.g., “blue laser” and/or “green laser”).
2. A larger amount of micromirrors is toggled to ensure that the laser light is directed to the spectrometer [see the green rays in Figure 3(b)].
3. After the spectral analysis, all micromirrors are switched back except those corresponding to the measured laser wavelengths.

## 6. Summary/Outlook

We presented here a novel concept to avoid dazzling of electro-optical sensors by continuous wave laser sources. It is based on a digital micromirror device (DMD) combined with wavelength multiplexing. This concept allows for selective spectral filtering within arbitrary areas of the sensor's field of view. Thus, all visual scene details will be preserved and not blacked out as in simple concepts without wavelength multiplexing.

To achieve automatic suppression, a control loop based on the recognition of dazzling incidents is necessary. In the case of a colour camera, the recognition algorithm analyses each single image to detect laser dazzling and to estimate the wavelength. The knowledge of the laser wavelength is essential in order to toggle the appropriate micromirrors of the DMD. The wavelength can be estimated directly from the RGB values of the pixels surrounding the overexposed regions of the camera image. The accuracy of the wavelength estimation depends on the specific colour response curves of the sensor and is in our case quite good for wavelengths up to 600nm.

The functionality of the concept was proven in field trials performed with different wavelengths. However, the current wavelength estimation method does not cover all conceivable situations and could suffer from wavelength ambiguities. An improvement of the wavelength estimation may be enabled, for example, by the use of a spectrometer in addition to the existing concept.
